# A decision support tool has similar high PrEP uptake and increases early PrEP persistence in adolescent girls and young women in South Africa: results from a randomized controlled trial

**DOI:** 10.1002/jia2.26154

**Published:** 2023-08-27

**Authors:** Connie Celum, Dominika Seidman, Danielle Travill, Christine Dehlendorf, Sanele Gumede, Kidist Zewdie, Whitney Wilson, Jennifer F. Morton, Jared M. Baeten, Deborah Donnell, Sinead Delany‐Moretlwe

**Affiliations:** ^1^ Departments of Global Health Medicine and Epidemiology University of Washington Seattle Washington USA; ^2^ Department of Obstetrics Gynecology & Reproductive Sciences University of California San Francisco San Francisco California USA; ^3^ Wits RHI University of the Witwatersrand Johannesburg South Africa; ^4^ Department of Family & Community Medicine University of California San Francisco San Francisco California USA; ^5^ Vaccine and Infectious Disease Division Fred Hutchinson Cancer Research Center Seattle Washington USA; ^6^ Department of Epidemiology University of Washington Seattle Washington USA; ^7^ Department of Global Health University of Washington Seattle Washington USA; ^8^ Gilead Sciences Inc. Foster City California USA

**Keywords:** choice, decision support tool, HIV prevention, pre‐exposure prophylaxis, South Africa, women

## Abstract

**Introduction:**

African adolescent girls and young women (AGYW) have high rates of HIV acquisition and are a priority population for HIV pre‐exposure prophylaxis (PrEP). PrEP implementation has been limited by AGYW's low perceived HIV risk and provider demands. A decision support tool (DST) with information about PrEP could improve clients’ risk perception, knowledge about PrEP, informed decision‐making and motivation to use PrEP based on their risk, facilitating PrEP delivery in primary healthcare (PHC) clinics.

**Methods:**

We designed MyPrEP, a client‐facing DST about PrEP and HIV prevention, with youth‐friendly information and images. The impact of the MyPrEP tool was assessed among HIV‐negative women aged 18–25 years presenting to a PHC clinic in Johannesburg, South Africa from March 2019 to 2020. AGYW were randomized by day to the DST or a general health website as the control condition. A clinician blinded to DST versus control allocation provided standard of care counselling about PrEP, offered PrEP, administered a questionnaire and conducted sexually transmitted infection testing. The primary outcome was PrEP initiation and the secondary outcome was PrEP persistence at 1 month, determined by pharmacy dispensation records.

**Results:**

Of 386 AGYW screened, 353 were randomized (DST *n* = 172, control *n* = 181) with a median age of 21 years (interquartile range [IQR] 20, 23) and 56% (199/353) attending the clinic for HIV testing, 46% (164/353) using contraception, 15% (53/353) using condoms consistently and 37% (108/353) with a curable sexually transmitted infection. PrEP was initiated by 97% in the DST group and 94% in the control group (OR 1.79; 95% confidence interval, CI = 0.79–1.53), of whom two‐thirds planned to continue PrEP until they decided if they liked PrEP. At 1 month, PrEP persistence was 19% in the DST and 10% in the control group (OR 1.97, 95% CI 1.08–3.69). Ninety‐nine percent randomized to the DST reported satisfaction with MyPrEP.

**Conclusions:**

Among AGYW attending a South African PHC clinic, PrEP uptake was >90% with two‐fold higher PrEP persistence at 1 month in those randomized to use the MyPrEP DST. Given the need for strategies to support PrEP implementation and improve low PrEP persistence among African AGYW, a PrEP DST warrants further evaluation.

## 1 INTRODUCTION

Adolescent girls and young women (AGYW) in Africa account for 25% of new HIV infections globally [1]. While pre‐exposure prophylaxis (PrEP) is highly effective when taken consistently [2], AGYW experience challenges with medication adherence [3, 4]. PrEP efficacy trials demonstrated that AGYW experienced barriers to PrEP initiation and persistence [5, 6], in part because HIV risk perception did not align with their sexual behaviour or sexually transmitted infections (STIs) [7, 8, 9]. Non‐stigmatizing methods that can be scaled within resource‐constrained healthcare services are needed to support AGYW to evaluate their HIV risk and motivate their PrEP use.

Client‐facing decision support tools (DSTs) have improved patient knowledge, involvement in shared decision‐making and accuracy of their perceived risk [10, 11]. DSTs provide information in a standardized way and aim to integrate patient values and preferences with clearly presented evidence about clinical options. These attributes may be beneficial in situations in which providers ask sensitive sexual behaviour questions and clients make decisions about potentially stigmatized services, such as PrEP, as well as when there are multiple options. DSTs may be beneficial in busy healthcare settings or when offering new services, when clinicians are less knowledgeable or confident in their counselling.

We developed a digital tablet‐based DST called MyPrEP to support AGYW in their HIV prevention decisions. We modelled the MyPrEP DST on My Birth Control, a DST used for contraception which demonstrated positive impacts on client experience and knowledge, and provider time allocation and ability to elicit client preferences [12, 13, 14]. We evaluated the effectiveness and acceptability of the MyPrEP DST on PrEP initiation and persistence in a randomized controlled trial among AGYW attending a primary healthcare (PHC) clinic in Johannesburg, South Africa.

## 2 METHODS

### 2.1 MyPrEP DST development

The development of the MyPrEP DST was based on iterative feedback from healthcare providers, community members and AGYW in South Africa and Kenya [15]. The DST was formatted on an electronic tablet and has since been formatted for web or smartphone access. MyPrEP DST presents available HIV prevention options in a neutral manner, and aims to support users’ informed decision‐making, including the option of not choosing PrEP. The DST is self‐directed with interactive content, begins with depictions of why AGYW choose to use HIV prevention, highlights attributes of HIV prevention options and presents a story of an AGYW who chooses to take PrEP. MyPrEP DST allows users to review modules with information about HIV prevention methods (PrEP, condoms, treatment as prevention and partner HIV testing), with in‐depth information on PrEP effectiveness, dosing, side effects and safety (Figure S1). MyPrEP DST concludes with a section addressing myths and facts about PrEP (https://mypreptool.org/en/).

### 2.2 Study design, setting and participants

From March 2019 to March 2020, MyPrEP DST was evaluated among English‐speaking, sexually active, HIV‐negative women, aged 18–25 years, who were not pregnant, presenting for health services at a PHC clinic in inner‐city Johannesburg. Over two‐thirds of Johannesburg residents access healthcare in PHC clinics, making this setting a key delivery point for PrEP [16]. The DST evaluation was nested within a demonstration project exploring models of PrEP delivery in South Africa and Kenya [17]. Although oral PrEP was approved in South Africa in 2016, the national programme was nascent in 2019–2020 with limited PrEP availability for AGYW in the district outside of this PHC. The study was reviewed and approved by the research ethics committees of the University of Witwatersrand, the University of Washington and the University of California, San Francisco. The study was registered through Clinicaltrials.gov (NCT03490058).

### 2.3 Study procedures

AGYW who presented for PHC services were invited to participate in the study and provided written consent for participation. Participants were randomized 1:1 by weekday to receive the MyPrEP DST or control (a general health education website). Randomization by day ensured that AGYW attending clinic together were randomized to the same intervention, minimizing cross‐arm peer influence that could influence results. Participants took approximately 10 minutes to review MyPrEP DST while waiting for their provider consultation.

Participants were subsequently seen by a nurse blinded to intervention allocation who responded to their primary reason for clinic attendance, offered HIV counselling and testing, and if they tested negative for HIV, a PrEP eligibility assessment and counselling per national guidelines [18]. Participants were also counselled about contraceptive options, and treated for STIs if symptomatic or if they had a positive urine‐based nucleic acid amplification test for *Neisseria gonorrhoeae* and *Chlamydia trachomatis* (Cepheid). Participants who accepted PrEP received 1 month of tenofovir disoproxil fumarate/emtricitabine tablets on the same day and were asked to return in 1 month for HIV testing and PrEP re‐supply.

Following administration of the control website or MyPrEP DST, participants responded to a brief interviewer‐administered questionnaire; the interviewer was separate from the clinical team to reduce socially desirable responses. As a measure of the quality of women's decision‐making about PrEP use, decisional conflict was assessed using a validated tool [19]. Data on socio‐demographics, behaviour, medical history, PrEP knowledge, attitudes about PrEP use, intentions to continue PrEP and acceptability of the information received were collected. HIV risk was assessed by the VOICE risk score which is predictive of HIV acquisition risk in African women [20].

### 2.4 Outcomes and analysis

The primary endpoint of the study, PrEP initiation, was defined as PrEP dispensation at enrolment. With 330 participants, the study had 80% power to detect ≥15% difference in PrEP initiation, assuming initiation by 60% in the control group and 75% in the DST group. The secondary endpoint, PrEP persistence at month 1 among those initiating PrEP, was selected due to high (40%–66%) early PrEP discontinuation rates observed in African young women [21, 22] and the impact of COVID‐19 lockdowns. PrEP persistence was determined from pharmacy records documenting a PrEP refill in 1 month with a 15‐day window, assuming that participants who did not return did not continue PrEP given the limited availability of PrEP in the public sector. PrEP initiation and PrEP continuation at month 1 were analysed by intention to treat by study arm, using generalized linear models with logit link(logistic regression). Potential correlation by day of randomization was accounted for by a sensitivity analysis using a mixed‐effect model with a random effect for those randomized on the same day. Descriptive statistics were used to summarize questionnaire responses by the study group. Decisional conflict scores by arm were compared using a generalized linear model with logit link. All analyses were done using R 4.0.

## 3 RESULTS

### 3.1 Participant characteristics

Of 386 women screened, 353 were randomized (Figure S2). Participants had a median age of 21 years (interquartile range [IQR] 20, 23), 97% (344/353) were not married, and 56% (199/353) were attending the clinic for HIV testing (Table 1). Sixty‐nine percent (142/353) had a previous pregnancy, 46% (164/353) were using contraception, of which injectable contraceptives were the most common method (52%, 86/164). With respect to HIV risk, 14% (49/353) had two or more sex partners, 15% (53/353) reported consistent condom use in the previous 3 months and 37% (108/353) had laboratory‐confirmed *N. gonorrhoeae* or *C. trachomatis*. One‐third of participants (102/353) reported substantial concern about acquiring HIV in the next 12 months. The median VOICE risk score was 6 (IQR 5,7) and few had previously used PrEP. Participant characteristics were balanced by a randomized group.

**Table 1:** Participant characteristics at enrolment, by study group

	General health website	My PrEP DST
	*N* = 181	*N* = 172
Characteristic	*n* (%) or median (IQR) or mean (SD)	*n* (%), median (IQR) or mean (SD)
Median age, years	21 (19, 24)	21 (20,23)
Not married	177 (98)	167 (97)
Reason for clinic visit (*N* = 350)		
HIV testing	102 (57)	97 (56)
Contraception	49 (28)	54 (31)
STI symptoms	1 (1)	0 (0)
Previous pregnancy	126 (70)	116 (67)
Consistent condom use, past 3 months	22 (12)	31 (18)
Two or more current sex partners	21 (12)	28 (16)
Known HIV‐positive partner	4 (2)	10 (6)
Currently contraceptive use	85 (47)	79 (46)
Current contraceptive method among users (*n* = 164)		
Injectable	47 (55)	39 (49)
Oral	19 (22)	13 (16)
Implant	8 (9)	12 (15)
Emergency contraception, condoms or other	13 (15)	13 (16)
Median VOICE risk score^a^	6 (5,7)	6 (5,7)
STI symptoms at visit	3 (1.7)	4 (2.0)
*C. trachomatis* ^b^	46/146 (32)	55/144 (37)
*N. gonorrhoeae* ^b^	12/146 (8)	11/144 (8)
Previous PrEP use	2 (1)	1 (1)
Concerned about HIV acquisition in the next 12 months		
A lot of worry	64 (35)	38 (22)
Some worry	53 (29)	40 (23)
Not worried	59 (33)	91 (53)
Concerned about PrEP side effects		
A lot of worry	24 (13)	15 (9)
Some worry	70 (39)	59 (34)
Not worried	82 (45)	94 (55)
Concerns about PrEP (can choose multiple)		
None	75 (41)	94 (55)
Pill size and taste	34 (19)	22 (13)
People should not take drugs unless sick	1 (1)	4 (2)
HIV stigma	31 (17)	35 (20)
Partner	23 (13)	18 (10)
Effect on fertility	28 (15)	19 (11)
Daily pill taking	22 (12)	19 (11)
PrEP use disclosure (can choose multiple)	141 (78)	138 (76)
Partner	63/141 (45)	54/138 (39)
Family member	60/141 (43)	69/138(50)
Friend	27/141 (19)	35/138 (25)

^a^ The modified VOICE risk score includes age<25; not married or living with partner; alcohol use in the past 3 months; partner does not provide financial/material support; and partner has other sex partners with a maximum score of 8.

^b^ Laboratory testing for *C. trachomatis* and *N. gonorrhoeae* was performed in 290 participants.

### 3.2 PrEP initiation and persistence

**Figure 1:**
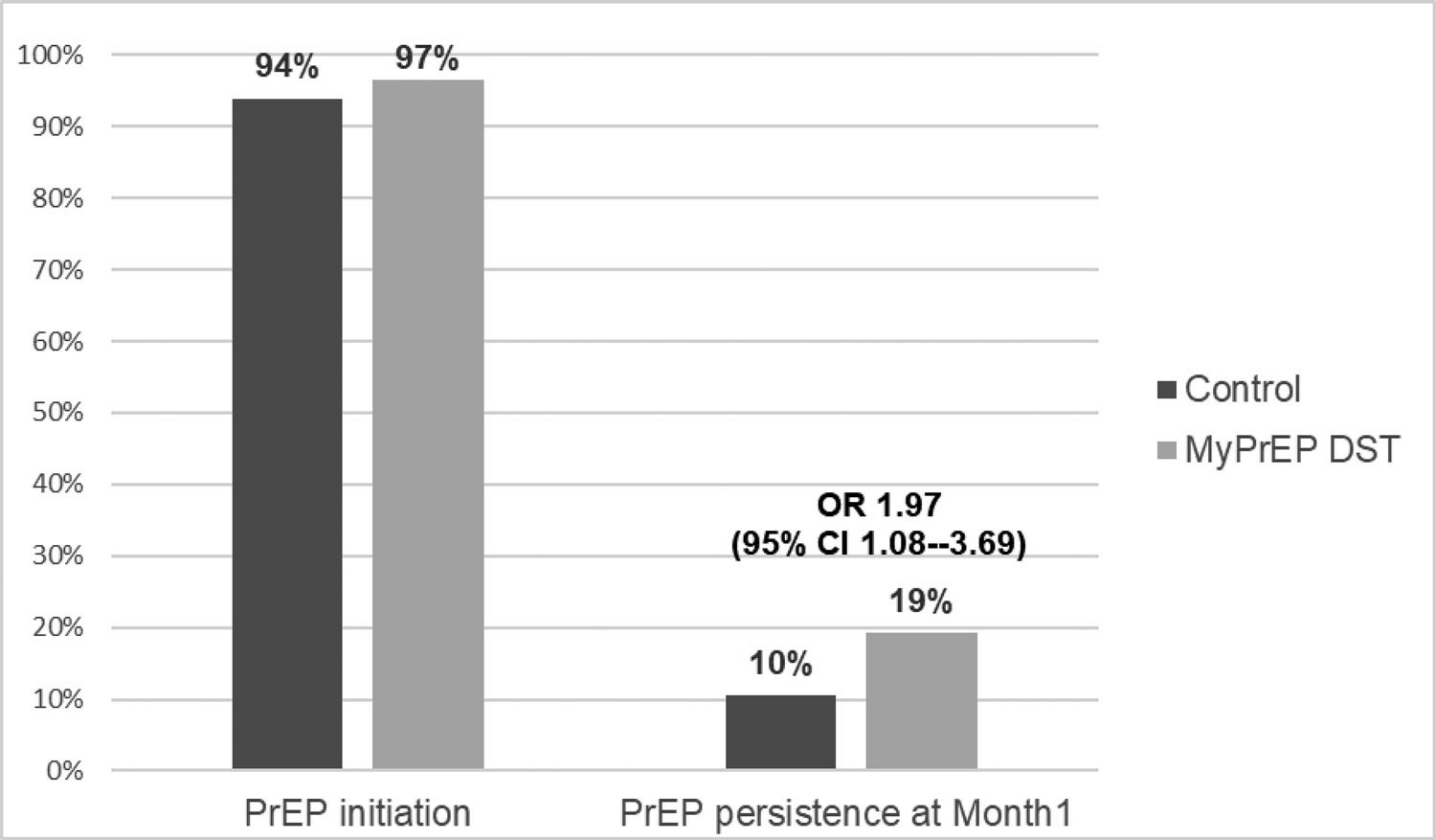
PrEP initiation and persistence, by study group. The histograms indicate the proportion of women randomized to the control condition (dark bars) and the My PrEP decision support tool (light bars) who received a PrEP prescription in the left histograms, and who attended their month 1 visit and received a PrEP refill in the right histograms.

PrEP initiation was 94% (170/181) in the control group and 97% (166/172) in the DST group (OR 1.79, 95% confidence intervals [CI] 0.79–5.30, *p* = 0.2; Figure 1). Two‐thirds of AGYW initiating PrEP planned to continue PrEP until they decided if they liked PrEP (Table 2), suggesting a need in this population to try out PrEP in order to decide about PrEP continuation.

**Table 2:** PrEP knowledge, attitudes and acceptability of information

	General health website	My PrEP DST
	*N* = 181	*N* = 172
Characteristic	*n* (%) or median (IQR) or mean (SD)	*n* (%) or median (IQR) or mean (SD)
Decisional conflict score^a^, mean (SD)	4.65 (9.8)	4.43 (8.8)
Decisional score^a^ >0	48 (27)	59 (35)
Believes PrEP will prevent HIV	176 (98)	164 (95)
Believes PrEP side effects self‐limiting	127 (71)	131 (76)
Believes PrEP prevents STIs other than HIV	1 (1)	2 (1)
I plan to take PrEP for as long as		
It takes me to decide if I like PrEP or not	120 (71)	119 (71)
I don't know	25 (15)	24 (14)
I am with my current partner	15 (9)	17 (10)
Until my partner has an HIV test	5 (3.)	3 (2)
I have more than one partner	3 (2)	4 (2)
Other	7 (4)	2.1 (1)
Satisfied with HIV information received	176 (99)	170 (98)
Information was useful	177 (99)	170 (99)
Received all the information that was needed	178 (99)	171 (99)
Information was easy to understand	175 (98)	171 (99)
Trusted the information received	172 (96)	166 (97)

^a^ Decisional conflict was estimated using an established scoring system that results in a range from 0 (no decisional conflict) to 100 (extremely high decisional conflict) [17].

Seventy‐three (21%) participants returned after 1 month (control group *n* = 33, DST *n* = 40). PrEP persistence at this visit was 10% (19/181) in the control and 19% (33/172) in the DST group (OR 1.97, 95% CI 1.08–3.69) (Figure 1). Results were similar in a mixed‐effects model that accounted for randomization by day.

### 3.3 PrEP knowledge and decision‐making

Decisional conflict was low; one‐third of participants indicated any decisional conflict with a non‐statistically significant trend of more decisional conflict reported in the DST group (35% vs. 27%, *p* = 0.1; Table 2). Neither participant knowledge nor satisfaction differed by study group (Table 2).

## 4 DISCUSSION

Among South African AGYW at risk for HIV randomized to a client‐facing digital DST or standard of care counselling, approximately 95% received an initial PrEP prescription. High PrEP acceptance may reflect the limited access to and novelty of PrEP during early implementation in South Africa and the desire of two‐thirds to decide about PrEP after trying it. The high PrEP interest limited our ability to evaluate the impact of the DST on PrEP uptake although PrEP continuation at 1 month was two‐fold higher than in the standard of care arm. Given the low persistence observed in PrEP projects among African AGYW [21, 22], the doubling of PrEP persistence at 1 month in a PHC is encouraging during the COVID‐19 lockdown and in terms of the potential of a client‐facing DST to facilitate informed PrEP decision‐making and improve PrEP persistence [17].

Women's expressed interest in a trial of PrEP use underlines that PrEP decision‐making is an ongoing process. This highlights the need for ongoing decision support, as individuals decide to sample one method, and then potentially another. The non‐significant trend towards more decision conflict among those randomized to the MyPrEP DST may reflect a more deliberative weighing of decisions. Evidence from contraceptive counselling has shown that early discontinuation of a method can be influenced by inadequate counselling related to the method and unanticipated or unacceptable side effects [23]. The DST may have helped AGYW continue PrEP through preparing them about possible side effects and understanding the importance of consistent pill‐taking to obtain prevention benefits. The lack of difference in participant knowledge and satisfaction by arm may relate to a high degree of satisfaction with the client−provider interaction rather than electronic tablet‐based information alone, a social desirability bias, or other biases in this population. Further analysis of qualitative data may provide additional insights, and future evaluations of the DST may investigate these outcomes further.

With new effective PrEP options becoming available, including the dapivirine vaginal ring and injectable cabotegravir, presentation of the range of PrEP options in a DST could help support women to make an initial informed choice about which PrEP formulation meets their needs and preferences, as well as assist them with decisions about switching PrEP methods. Further evaluation of the DST in the setting of expanded options, and the use of the DST to support persistence and switching of PrEP methods will be critical to understanding how the DST can be best utilized to support women's HIV prevention decision‐making.

The limitations of this study are generalizability as the study was conducted among English speakers in one PHC clinic in South Africa in which the research context could have influenced the high PrEP uptake. Participant retention was low and influenced by common health systems challenges in this setting, including contraceptive stockouts, intermittent water and electricity interruption, civil unrest, as well as COVID‐19 lockdowns. Since a blood sample for tenofovir levels as an objective measure of PrEP adherence was not obtained at the month 1 visit, it is not possible to compare PrEP adherence by study arm. Our findings could have been due to chance and additional research is warranted with longer follow‐up. Despite these limitations, the study was implemented in a real‐world setting early in the rollout of the South African PrEP programme. The DST is a brief client‐level intervention which was developed with significant end‐user input and could be used in a waiting room or virtually, making it feasible to scale up in diverse settings.

## 5 CONCLUSIONS

The use of a PrEP DST for South African AGYW is a promising strategy to support PrEP use. Web availability of DSTs for remote use or in clinic waiting rooms may facilitate expanded use of DSTs, even in low‐resource settings as mobile and internet coverage in the region expands. The COVID‐19 pandemic has accelerated demand for remote clinical consultations; DSTs, such as MyPrEP, may facilitate HIV prevention service provision outside of clinical settings in the future. As new PrEP methods and multipurpose technologies become available, information about new effective PrEP formulations can be readily incorporated into the MyPrEP DST, potentially facilitating informed decision‐making by AGYW about HIV prevention choices that best meet their needs.

## COMPETING INTERESTS

CC has been a scientific advisor to Gilead Sciences and Merck and served as an expert witness for Gilead. SD‐M has been a scientific advisor to Merck Sharp Dohme. JMB is an employee of Gilead Sciences, outside of the present work.

## AUTHORS’ CONTRIBUTIONS

CC, SD‐M, JMB, DS and CD designed the research study. DT and SG conducted the research. KZ and DD performed the analysis. CC, DS and SD‐M wrote and revised the manuscript. All authors have read and approved the final manuscript.

## FUNDING

This work was supported by a grant from the National Institutes of Mental Health (R01MH114544) and from USAID (OAA‐A‐15‐00034).

## Supporting information


**Figure S1**: Screen images of My PrEP Decision Support Tool.
**Figure S2**: Screening, enrolment and randomization by study arm.

## Data Availability

Individual participant data that underlie the results reported in this article, after de‐identification, are available, beginning after publication, as well as the study protocol, and data dictionary, for researchers with a methodologically sound proposal.
