# Supplementary material for: A decision support tool has similar high PrEP uptake and increases early PrEP persistence in adolescent girls and young women in South Africa: results from a randomized controlled trial
Source: J Int AIDS Soc. 2023 Aug 27;26(8):e26154. doi: 10.1002/jia2.26154 (PMC10460672; doi:10.1002/jia2.26154)
Supplement: Supplementary file 1 — Figure S1: Screen images of My PrEP Decision Support Tool. Figure S2: Screening, enrolment and randomization by study arm. [file JIA2-26-e26154-s001.docx]

**Supplement**

**Figure 1: Screen images of My PrEP Decision Support Tool**

**
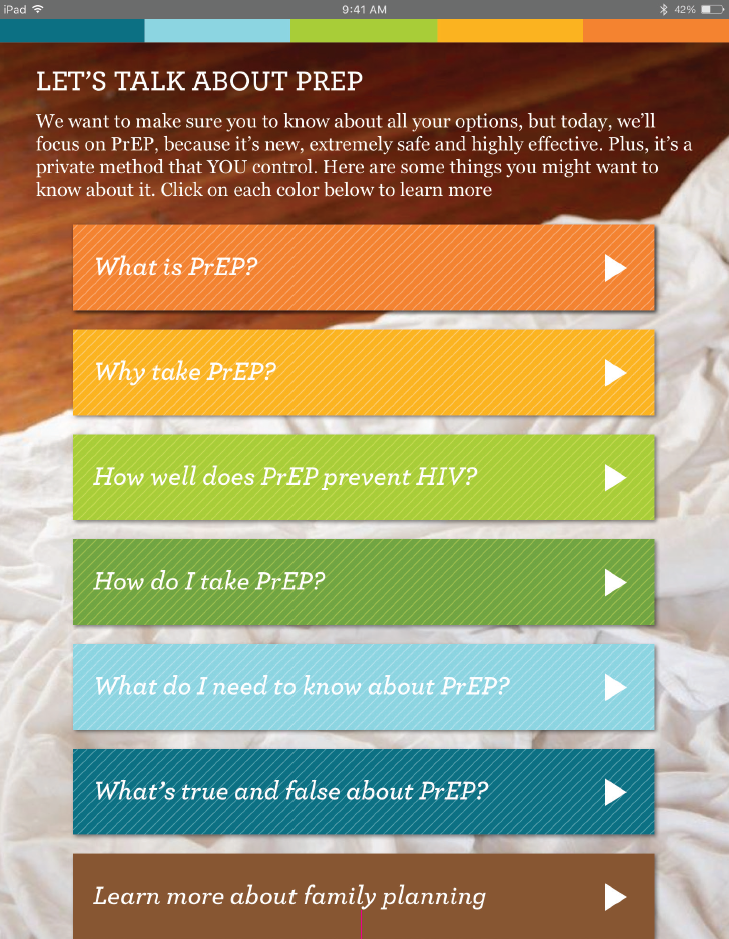
**

**Legend for Supplemental Figure 1a:** Screen from MyPrEP decision support tool that is interactive and enables client to learn about rationale, efficacy, user considerations, and misconceptions about PrEP, followed by a brief module about family planning.

**Figure 1b:** User information about daily oral emtricitabine-tenofovir PrEP for HIV prevention. The user messages are designed to be simple and informative without statistics or complex medical terminology.

**
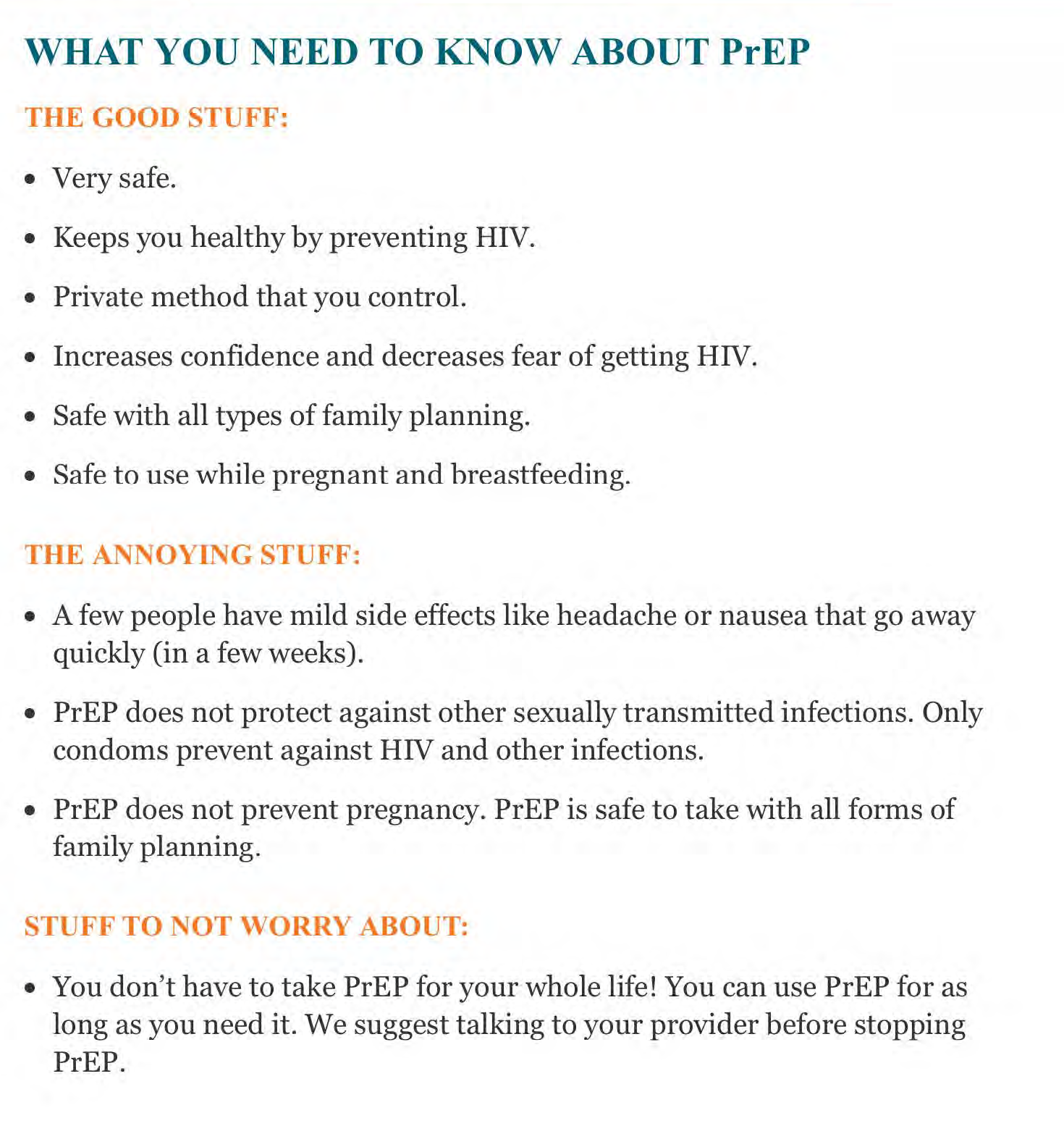
**

**Figure 2: Screening, enrolment and randomization by study arm**
